# Identifying sexual differentiation genes that affect Drosophila life span

**DOI:** 10.1186/1471-2318-9-56

**Published:** 2009-12-09

**Authors:** Jie Shen, Daniel Ford, Gary N Landis, John Tower

**Affiliations:** 1 Molecular and Computational Biology Program, Department of Biological Sciences, University of Southern California, Los Angeles, CA 90089-2910, USA

## Abstract

**Background:**

Sexual differentiation often has significant effects on life span and aging phenotypes. For example, males and females of several species have different life spans, and genetic and environmental manipulations that affect life span often have different magnitude of effect in males versus females. Moreover, the presence of a differentiated germ-line has been shown to affect life span in several species, including *Drosophila *and *C. elegans*.

**Methods:**

Experiments were conducted to determine how alterations in sexual differentiation gene activity might affect the life span of *Drosophila melanogaster. Drosophila *females heterozygous for the *tudor[1]* mutation produce normal offspring, while their homozygous sisters produce offspring that lack a germ line. To identify additional sexual differentiation genes that might affect life span, the conditional transgenic system Geneswitch was employed, whereby feeding adult flies or developing larvae the drug RU486 causes the over-expression of selected UAS-transgenes.

**Results:**

In this study germ-line ablation caused by the maternal *tudor[1]* mutation was examined in a long-lived genetic background, and was found to increase life span in males but not in females, consistent with previous reports. Fitting the data to a Gompertz-Makeham model indicated that the maternal *tudor[1]* mutation increases the life span of male progeny by decreasing age-independent mortality. The Geneswitch system was used to screen through several UAS-type and EP-type P element mutations in genes that regulate sexual differentiation, to determine if additional sex-specific effects on life span would be obtained. Conditional over-expression of *transformer *female isoform (*traF) *during development produced male adults with inhibited sexual differentiation, however this caused no significant change in life span. Over-expression of *doublesex *female isoform (*dsxF) *during development was lethal to males, and produced a limited number of female escapers, whereas over-expression of *dsxF *specifically in adults greatly reduced both male and female life span. Similarly, over-expression of *fruitless *male isoform *A *(*fru-MA*) during development was lethal to both males and females, whereas over-expression of *fru-MA *in adults greatly reduced both male and female life span.

**Conclusion:**

Manipulation of sexual differentiation gene expression specifically in the adult, after morphological sexual differentiation is complete, was still able to affect life span. In addition, by manipulating gene expression during development, it was possible to significantly alter morphological sexual differentiation without a significant effect on adult life span. The data demonstrate that manipulation of sexual differentiation pathway genes either during development or in adults can affect adult life span.

## Background

The disposable soma theory suggests that aging occurs because there is a selection pressure to assign limited biological resources to short-term survival, growth, and reproduction, rather than long-term survival [1,2]. Consistent with this idea, several studies have suggested that there may exist a trade-off between reproduction and life span. For example, in humans, longer life span has been correlated with a smaller number of offspring [3], but see also [4,5]. In *C. elegans*, elimination of reproduction by ablation of the germ line extended life span by up to +60% [6]. This effect was attributed to increased activity of the insulin/IGF1-like signaling (IIS) pathway target transcription factor DAF-16 in the gastrointestinal tract, caused by reduced hormonal signaling from the gonad to the intestine [7].

A trade-off between life span and reproduction does not appear to be obligatory, because it is possible in certain instances to increase life span in *C. elegans *and *Drosophila *without causing a decrease in reproduction [8,9]. One possibility is that it is sexual differentiation, rather than reproduction *per se*, that exerts a cost on life span [10].

In *Drosophila *males and females often respond differently to genetic or environmental interventions that extend life span. For example, reduced IIS, dietary restriction and virginity each caused greater increase in life span in females than in males [11,12]. Similarly, over-expression of mutant forms of *p53 *preferentially increased female life span [13,14]. Possible mechanisms for sex-specific life spans include asymmetric inheritance of sex chromosomes, genetic differences between the sexes, differences in physiology and behavior, maternal effects, and sexual selection, sexual conflict, or sex linkage [10,11,15].

In *Drosophila*, elimination of germ cells (GCs) by forced expression of the differentiation gene *bam *in late development or adulthood was found to increase median life span by +14% to +78% in males, and by +23% to +100% in females [16]. The elimination of *Drosophila *GCs was found to modulate IIS, by increasing nuclear localization of the *Drosophila *homolog of DAF-16 (called dFoxo), and by increasing the expression of *Drosophila insulin*-*like peptides *(*dilps*) [16]. The *Drosophila *maternal effect genes *germ cell-less *and *tudor *are necessary for the formation of the germ line in offspring [17]. Interestingly, in another recent study, it was concluded that elimination of *Drosophila *GCs using *germ cell-less *and *tudor *mutations might not extend life span [18], and the reason for the difference in results in these previous studies may be differences in the timing of germ cell ablation relative to fly development. Here the maternal *tudor[1]* mutation was tested in a particularly long-lived genetic background, and was found to increase the life span of male offspring, but to have neutral or negative effects on female life span.

**Figure 1:**
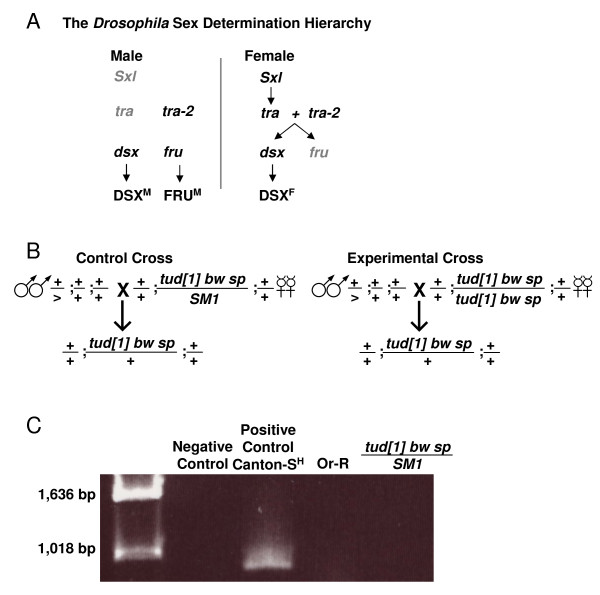
***Drosophila *sex determination hierarchy and the *tudor *mutation**. A) The *Drosophila *somatic sex determination hierarchy. The *X *to autosome ratio causes *Sxl *to be "off" in males (indicated in gray color) and to be "on" in females. In males, lack of SXL and TRA activity leads to expression of FRU^M ^and DSX^M ^. In females, TRA and TRA2 together direct splicing of downstream targets to produce DSX^F ^, and *fru *P1 transcripts that are not translated in females. B) Crossing scheme to produce germ line-ablated flies using *tudor[1]*. For the experimental group, *tudor *homozygote females were crossed to wild type Oregon-R males to generate progeny lacking a germ line. For the control group, *tudor *heterozygote females were crossed to Oregon-R males to generate progeny containing a normal germ line. Both experimental and control groups have the same chromosomal composition. B) Test for *Wolbachia*. The *Wolbachia *16S rDNA sequences were amplified by PCR from the indicated *Drosophila *lines and controls, and the presence or absence of *Wolbachia*-specific PCR products was determined by gel electrophoresis and ethidium bromide staining.

In the *Drosophila *soma, sexual differentiation is determined by the *X *chromosome to autosome (*X:A*) ratio and the on/off status of the master regulatory gene *Sex-lethal *(*Sxl*) (Figure 1A), however cell-cell interactions also play a role [19]. Sexual differentiation in the *Drosophila *germ line is determined by germ line-autonomous signals, as well as the interactions between the germ line and the soma [20-22]. Genes in the somatic sex determination hierarchy play important roles but are not the primary regulators in germ line sexual differentiation. For example, *Sxl *promotes oogenic development in female germ cells, but not in male germ cells.

To test if additional alterations in sexual differentiation might affect fly life span, the Geneswitch system was used to screen through several UAS-type and EP-type P element mutations in genes that regulate sexual differentiation. This approach allows genes to be over-expressed either during *Drosophila *larval development, or specifically in the adult stages. The *Drosophila *sex determination hierarchy consists of pre-mRNA splicing factors encoded by the genes *Sex-lethal *(*Sxl*), *transformer *(*tra*), and *transformer-2 *(*tra*-*2) *[23] (Figure 1A). In females (sex chromosome composition *X/X*), the ratio of *X *chromosomes to autosomes results in the production of SXL protein, which directs the pre-mRNA splicing of *tra *transcripts. The TRA and TRA-2 proteins together direct the splicing of pre-mRNAs for the transcription factor genes *doublesex *(*dsx) *and *fruitless *(*fru*), such that females produce the female form of the *doublesex *protein (DSX^F ^), and no sex-specific forms of Fruitless protein. In males (sex chromosome composition *X/Y*), the SXL and TRA products are not produced, which results in production of the male form of *doublesex *(DSX^M ^), and the male form of Fruitless protein (FRU^M ^). The DSX and FRU transcription factors then direct sex-specific differentiation of tissues and the potential for sex-specific behaviors. The data presented here demonstrate that the *Drosophila *sexual differentiation pathway can act during development and in adults to affect longevity.

## Methods

### Drosophila Strains

The UAS-transgene strains were generated by various laboratories, and stocks were obtained from Michelle Arbeitman at USC: The strain *y w; P{UAS-fruMA}*[7];+ is described in [24]. The strain *w; P{UAS-fru-IR}/CyO; P{UAS-fru-IR} *is described in [25]. The strain *w; P{UAS-dsxM}/CyO, GFP; *+ is described in [26]. The strain *w; +; P{UAS-dsxF}/TM3, Sb *was generated by Ken Burtis and is described in [27]. The strain *w; P{UAS-tra} [20J7]; *+ is described in [28]. The strains *w; +; P{dsx-XP} [d09625] *and *w; P{tra2-XP} [d10032]; + *are described in [29]. The strain *y[1] w [67c23] P{EPgy2}Sxl [EY06108]/FM6B *was obtained from Bloomington *Drosophila *stock center. The *tudor[1]* mutant strain [30] was also obtained from Bloomington *Drosophila *stock center. The genotypes of all strains are presented in Table 1. For experiments involving *tudor[1]*, the experimental group consisted of the progeny of *tudor[1]* homozygous females crossed to Oregon R wild-types males, while the control group consisted of the offspring of *tudor[1]* heterozygotes crossed to Oregon R males. This resulted in control and experimental groups with the same chromosomal composition, however the experimental group lacks a germ line due to the maternal effect of homozygous *tudor[1]* (Figure 1B). The tissue-general Geneswitch driver line *Act-GS-255B *contains multiple copies of a construct in which the tissue-general *actin5C *promoter drives expression of the Geneswitch protein, and has previously been described and characterized [13,31]. *Act-GS-255B *virgins were used in the crosses with males of other lines, unless the UAS insertion of the sex differentiation gene or the EP (XP) insertion was on the *X *chromosome, in which case the cross direction was reversed.

**Table 1:** Starting stocks.

*St#*	*Genotype*	*Notes*	*Abbreviation*
1	*w [1118]; Act-GS-255B;+*	*Tissue-general Geneswitch driver*	*255B*
2	*y w; P{UAS-fruMA}[7]; +*	*UAS-fru male A isoform*	*fruMA*
3	*w; P{UAS-fru-IR}/CyO; P{UAS-fru-IR} *	*UAS-fru RNAi*	*fruIR*
4	*w; P{UAS-dsxM}/CyO, GFP; +*	*UAS-dsx male isoform*	*dsxM*
5	*w P{UAS-tra2-IR} [61A]; +; P{UAS-tra2-IR} [82A]*	*UAS-tra2 RNAi*	*tra2IR*
6	*w [1118]; +; +*	*Injection strain control*	
7	*Oregon R (+; +; +)*	*wild type control*	
9	*w; +; P{UAS-dsxF}/TM3, Sb*	*UAS-dsx female isoform*	*dsx*
10	*w; P{UAS-tra} [20J7]; *+	*UAS-tra*	*tra*
11	*y[1] w [67c23] P{EPgy2}Sxl [EY06108]/FM6B*	*EP-sxl*	*sxl*
14	*w; +; P{dsx-XP} [d09625]*	*XP-dsx*	*dsx*
15	*w; P{tra2-XP} [d10032]; *+	*XP-tra2*	*tra2*
16	*+; tud[1] bw sp/SM1; +*	*tudor[1] mutant*	

### Fly Culture

*Drosophila *culture and life span assays were performed essentially as previously described [31]. Briefly, *Drosophila *were cultured on a dextrose, agar, yeast, and cornmeal media [32], and adults were maintained at twenty-five flies per vial. Survival assays were performed at 25°C. Every two days, flies were transferred to new vials, and the number of deaths was recorded. The drug RU486 (Mifepristone, Sigma) was dissolved in ethanol (100%) to make a stock solution at 3.2 mg/ml. For adult feeding, 50 ul of RU486 stock solution was added to each vial to produce a final concentration of ~160 ug/ml; 50 ul ethanol was added to the control vials. For larval feeding, 0.5 ml of 3.2 mg/ml RU486 stock solution was added to each bottle, whereas 0.5 ml ethanol was added to controls. Vials and bottles were covered with cheesecloth and allowed to dry overnight to allow the ethanol to evaporate.

### Wolbachia Test

Total DNA was extracted from ten male and ten female flies of each line using the ZR Genomic DNA Kit II (Zymo Research). The *Drosophila *DNA was then used as template for PCR amplification with *Wolbachia*-specific primers, and the products were fractionated on an agarose gel and stained with ethidium bromide [32,33].

### Phenotype Characterization

UAS-*tra *males were crossed to *Act-GS-255B *virgins, and the progeny were cultured on food with drug (+RU486) to drive the over expression of *tra *during development, or cultured on food with ethanol as the control. Male and female external genitalia, abdomenal pigmentation patterns, and male sex combs were photographed using a Leica MZ FLIII fluorescence stereomicroscope. Or-R and *w [1118] *males were also crossed to *Act-GS-255B *virgins, and the progeny were cultured on food supplemented with drug (+RU486) or with ethanol only, as the controls.

### Statistical Analysis

Mean, standard deviation, median, percent change in mean, percent change in median, and log rank *p *value were calculated using R 2.6.2. Analysis of mortality rate was performed using the *WinModest *statistical package [34]. The best fit model was determined by the "Model Comparison" option and confirmed by other additional options of WinModest software. In the Gompertz-Makeham model, the increase of mortality (μ_*x *_) with age (*x*) is expressed as: μ_*x *_= *a*e^*bx *^+c, where the constant *a *is the initial mortality rate, *b *is the rate of exponential increase in mortality, and c is the age-independent mortality. WinModest assumes that the observation on the cohorts is continuous and ages at death are exact. Fly deaths were recorded every other day. Therefore, age was divided by two before being input into *WinModest*. The output age was then multiplied by two to compensate for the initial division. Without dividing the age by two and then multiplying the output by two, the trend-lines based on the parameters (a, b, c) would be too low, and would not perfectly match the mortality data points. The natural logarithm of mortality (Ln (μ_*x *_)) for each time point was calculated by the *WinModest *statistical software, based on: P_*x *_= N_*x + 1 *_/N_*x *_, μ_*x *_= -Ln(P_*x *_). Parameters (a, b, c) were also calculated by the *WinModest *statistical package, based on a likelihood ratio test. The full model (*a*e^*bx *^+ c) was plotted, and the Gompertz-only component (μ_*x *_) was used to build the decomposed survival curves, from the reverse calculation of μ_*x *_: μ_*x *_= *a*e^*bx *^, . For the decomposed survival curves, any value below 0.5% survival was considered to be the final data point.

## Results

**Figure 2:**
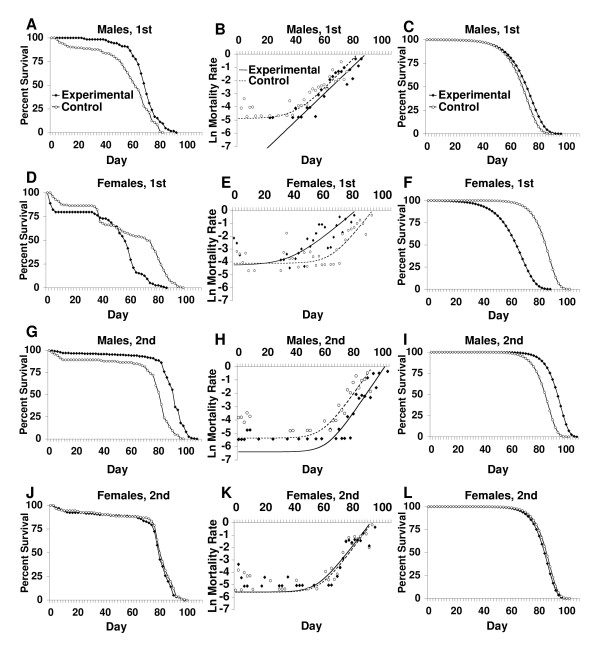
**Life span assays and mortality rate analysis for germ line-ablated *Drosophila***. A) Males lacking the germ line. The assay consisted of 125 flies for the experimental group and 122 flies for the control group. B) Mortality rate (Gompertz-Makeham model) of the germ line-ablated and control male groups. C) Redrawn survival curve for males with age-independent mortality removed. D) Females lacking the germ line. The assay consisted of 118 flies for the experimental group, and 124 flies for the control group. E) Mortality rate (Gompertz-Makeham model) of the germ line-ablated and control female groups. F) Redrawn survival curve for females with age-independent mortality removed. G) Males lacking the germ line, repeat assay. The assay consisted of 238 flies for the experimental group and 139 flies for the control group. H) Mortality rate (Gompertz-Makeham model) of the germ line-ablated and control male groups. I) Redrawn survival curve for males with age-independent mortality removed. J) Females lacking the germ line, repeat assay. The assay consisted of 233 flies for the experimental group and 175 flies for the control group. K) Mortality rate (Gompertz-Makeham model) of the germ line-ablated and control female groups. L) Redrawn survival curve with age-independent mortality removed.

Female flies heterozygous or homozygous for the *tudor[1]* mutation were crossed to Oregon-R wild-type male flies to produce control offspring and offspring lacking the germ line, respectively (Figure 1B). In the first experiment the life span of control and germ line-ablated flies was measured using cohorts of ~125 flies each. For the germ line-ablated male flies the mean and median life span was increased by +19.9% and +12.9%, respectively, relative to controls, whereas in contrast female life span was significantly decreased (Table 2). To determine if these results were reproducible, the experiment was repeated with cohorts sizes of ~240 flies. Male mean and median life span was again found to be increased by +19.97% and +12.2%, respectively, whereas female life span was not altered. Plots of percent survival versus time indicated that there was also significant early mortality in several of the cohorts (Figure 2A, D, G, J). Consequently, the *Winmodest *statistical package was used to control for early mortality and to analyze the survival data in greater detail. The data was fitted to a Gompertz-Makeham model where the constant *a *is the initial mortality rate, *b *is the rate of exponential increase in mortality, and c is the age-independent mortality (Figure 2B, E, H, K). The values of a, b and c were calculated based on a likelihood ratio test (Table 3). Re-plotting of the fitted data using only the Gompertz term yields a decomposed survival curve consisting of only the age-dependent mortality (Figure 2C, F, I, L). The data indicate that the increase in mean life span of germ line-ablated males relative to controls can be attributed to a decrease in age-independent mortality (rate constant *c*), while the initial mortality rate (rate constant *a*) and mortality rate increase with time (rate constant *b*) were not significantly affected (Table 3). While the initial experiment indicated a decrease in the life span of germ line-ablated females relative to controls, this result was not reproduced in the larger cohorts, where there was no significant difference in any of the mortality rate parameters between germ line-ablated females and controls.

**Table 2:** Statistical analysis of *tudor *life span assays.

Group	N	**Mean**^*a *^	Median	% Change in Mean	%Change in Median	Log Rank p Value
*Exp 1*						
Mutant Males	125	68.45 ± 11.37	70	19.91	12.90	**4.25E-05**
Control Males	122	57.08 ± 20.28	62			
Mutant Females	118	47.36 ± 24.50	56	-20.99	-23.29	**5.33E-10**
Control Females	124	59.94 ± 28.03	73			
*Exp 2*						
Mutant Males	238	87.41 ± 18.03	92	19.97	12.20	**1.00E-10**
Control Males	139	72.86 ± 25.01	82			
Mutant Females	233	74.33 ± 22.01	80	-2.04	0.00	0.121
Control Females	175	75.87 ± 21.78	80			

^*a *^Mean life span, days +/- SD.

**Table 3:** Parameters for Gompertz-Makeham model and likelihood ratio test results.

	Parameters	Mutant	Control	**chi**^2 ^	df	p Value	**chi**^2 ^	df	p Value
*Tudor Exp 1*		one parameter compared at each time							
Males							Both a and b are constrained		
	a	1.00 × 10^-4 ^	7.00 × 10^-5 ^	0.13	1	0.724			
	b	2.07 × 10^-1 ^	2.29 × 10^-1 ^	0.73	1	0.392			
	c	2.06 × 10^-9 ^	7.52 × 10^-3 ^	17.65	1	**< 0.001**	23.76	1	**< 0.001**
Females							c is constrained		
	a	3.20 × 10^-4 ^	1.04 × 10^-6 ^	13.23	1	**< 0.001**	13.34	1	**< 0.001**
	b	1.93 × 10^-1 ^	2.91 × 10^-1 ^	5.84	1	**0.016**	5.47	1	**0.019**
	c	1.43 × 10^-2 ^	1.66 × 10^-2 ^	0.37	1	0.541			
*Tudor Exp 2*									
Males							Both a and b are constrained		
	a	8.38 × 10^-8 ^	6.03 × 10^-7 ^	2.60	1	0.107			
	b	3.19 × 10^-1 ^	3.06 × 10^-1 ^	0.23	1	0.633			
	c	1.67 × 10^-3 ^	4.67 × 10^-3 ^	7.98	1	**0.005**	18.46	1	**< 0.001**
Females									
	a	6.40 × 10^-7 ^	3.46 × 10^-7 ^	0.33	1	0.565			
	b	3.07 × 10^-1 ^	3.17 × 10^-1 ^	0.15	1	0.694			
	c	3.76 × 10^-3 ^	3.69 × 10^-3 ^	0.00	1	0.949			

*Wolbachia *are gram-positive bacteria that are transmitted in *Drosophila *through maternal inheritance [35]. These bacteria are capable of altering life span, and can potentially result in a false positive for life span extension [8,36]. Therefore, the presence of *Wolbachia *was assayed by PCR using primers specific for the *Wolbachia *16S RNA genes, and the lines used in this experiment were found not to be infected (Figure 1C).

**Figure 3:**
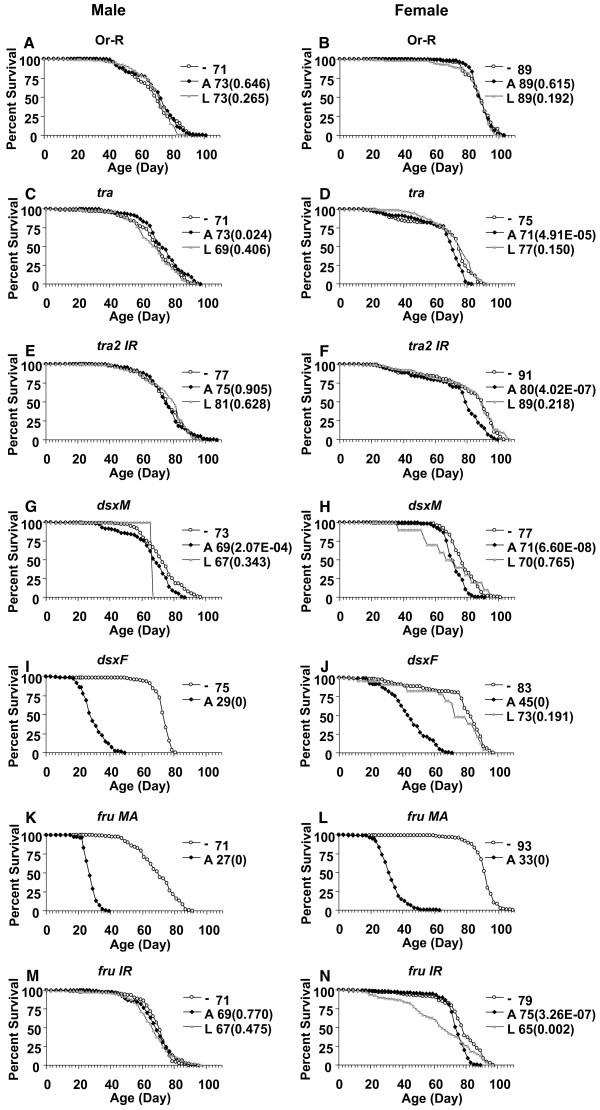
**Effect of sex differentiation pathway gene mis-expression on survival of male and female adult flies**. Sexual differentiation pathway genes or RNAi constructs were over-expressed either during larval development ("L"; gray triangles) or in adults ("A"; solid squares). Open circles represent the no-drug control ("-"). Survival curves are plotted as a function of adult age in days. Median life span and *p *value for log rank test are indicated in parentheses for each cohort. (A, B) Control flies (progeny of driver crossed to Or-R wild type). (C, D) *tra*. (E, F) *tra2-IR*. (G, H) *dsxM*. (I, J) *dsxF*. (K, L) *fruMA*. (M, N) *fru-IR*.

To begin to ask if other alterations in sexual differentiation could affect life span, the Geneswitch system was used to cause over-expression or inhibition of several genes involved in the sex-determination pathway. Expression of the Geneswitch transcription factor was driven with the cytoplasmic actin *Actin5C *promoter, using transgenic line *Act-GS-255B*. Feeding animals the drug RU486/Mifepristone either during larval development or as adults causes activation of the Geneswitch transcription factor, which then binds to UAS sites in target promoters and activates expression of the gene of interest or the RNAi construct. To control for any effects of the drug itself, the *Act-GS-255B *line was crossed to Oregon R wild type strain to generate progeny containing *Act-GS-255B *but no target construct. In these control flies, the drug treatment generally had no effect on life span, with the exception of two experiments where life span of adult females was reduced by -2% to -5% (Figure 3A, B; Table 4). The *Act-GS-255B *line was also crossed to the *w [1118] *injection strain to generate another control. In these control flies, the drug treatment had no effect on male life span, and had a slight effect on female life span ranging from -5% to +5% (Table 4). However, because these changes in controls were small in magnitude and were not always observed, we interpret these changes as being within the background of the assay. The drug treatment during development caused no visible alterations in sexual differentiation of the flies (data not shown).

**Table 4:** Life span data with means, standard deviations, medians, percent change in mean and median, and log rank p value.

Cross MxF	RU486	*Genotype*	Sex	N	**Mean**^*a *^	Median	%Change in Mean	%Change in Median	Log Rank p Value
*Exp 1*									
7-1	-	*w/Y; 255B/+; +*	M	122	68.85 ± 14.18	71	---------	---------	---------
7-1	A	*w/Y; 255B/+; +*	M	112	70.64 ± 13.48	73	2.6	2.82	0.646
7-1	L	*w/Y; 255B/+; +*	M	145	69.63 ± 12.16	73	1.14	2.82	0.265
7-1	-	*w/+; 255B/+; +*	F	121	87.78 ± 8.83	89	---------	---------	---------
7-1	A	*w/+; 255B/+; +*	F	117	88.56 ± 6.85	89	0.89	0	0.615
7-1	L	*w/+; 255B/+; +*	F	120	85.96 ± 11.95	89	-2.07	0	0.192
10-1	-	*w/Y; 255B/tra; +*	M	122	68.60 ± 15.83	71	---------	---------	---------
10-1	A	*w/Y; 255B/tra; +*	M	118	73.10 ± 13.38	73	6.56	2.82	**0.024**
10-1	L	*w/Y; 255B/tra; +*	M	123	67.54 ± 14.05	69	-1.54	-2.82	0.406
10-1	-	*w; 255B/tra; +*	F	122	68.75 ± 18.34	75	---------	---------	---------
10-1	A	*w; 255B/tra; +*	F	122	66.51 ± 15.22	71	-3.27	-5.33	**4.91E-05**
10-1	L	*w; 255B/tra; +*	F	127	73.03 ± 13.07	77	6.22	2.67	0.15
1-5	-	*w tra2IR/Y;255B/+;tra2IR/+*	M	119	74.82 ± 13.69	77	---------	---------	---------
1-5	A	*w tra2IR/Y;255B/+;tra2IR/+*	M	120	75.33 ± 12.55	75	0.69	-2.6	0.905
1-5	L	*w tra2IR/Y;255B/+;tra2IR/+*	M	122	76.28 ± 13.32	81	1.96	5.19	0.628
1-5	-	*w tra2IR/w;255B/+;tra2IR/+*	F	119	80.43 ± 22.10	91	---------	---------	---------
1-5	A	*w tra2IR/w;255B/+;tra2IR/+*	F	128	74.20 ± 19.64	80	-7.74	-12.09	**4.02E-07**
1-5	L	*w tra2IR/w;255B/+;tra2IR/+*	F	127	82.20 ± 20.76	89	2.21	-2.2	0.218
4-1	-	*w/Y; 255B/dsxM; +*	M	120	72.39 ± 12.77	73	---------	---------	---------
4-1	A	*w/Y; 255B/dsxM; +*	M	118	66.14 ± 13.80	69	-8.64	-5.48	**2.07E-04**
4-1	L	*w/Y; 255B/dsxM; +*	M	1	67.00 ± NA	67	-7.45	-8.22	0.343
4-1	-	*w;255B/dsxM; +*	F	118	77.20 ± 10.40	77	---------	---------	---------
4-1	A	*w;255B/dsxM; +*	F	121	71.91 ± 6.83	71	-6.86	-7.79	**6.60E-08**
4-1	L	*w; 255B/dsxM; +*	F	10	69.60 ± 18.21	70	-9.85	-9.09	0.765
9-1	-	*w/Y; 255B/+; dsxF/+*	M	115	72.23 ± 10.20	75	---------	---------	---------
9-1	A	*w/Y; 255B/+; dsxF/+*	M	108	29.78 ± 7.60	29	-58.77	-61.33	**0**
9-1	L	*w/Y; 255B/+; dsxF/+*	M	0	---------	---------	---------	---------	---------
9-1	-	*w; 255B/+; dsxF/+*	F	117	77.05 ± 19.31	83	---------	---------	---------
9-1	A	*w; 255B/+; dsxF/+*	F	99	44.06 ± 13.39	45	-42.82	-45.78	**0**
9-1	L	*w; 255B/+; dsxF/+*	F	23	71.57 ± 21.55	73	-7.12	-12.05	0.191
2-1	-	*w/Y; 255B/fruMA; +*	M	118	69.27 ± 12.50	71	---------	---------	---------
2-1	A	*w/Y; 255B/fruMA; +*	M	126	27.83 ± 4.03	27	-59.83	-61.97	**0**
2-1	L	*w/Y; 255B/fruMA; +*	M	0	---------	---------	---------	---------	---------
2-1	-	*w/y w; 255B/fruMA; +*	F	113	90.34 ± 10.49	93	---------	---------	---------
2-1	A	*w/y w; 255B/fruMA; +*	F	123	32.83 ± 7.76	33	-63.66	-64.52	**0**
2-1	L	*w/y w; 255B/fruMA; +*	F	0	---------	---------	---------	---------	---------
3-1	-	*w/Y; 255B/fruIR; fruIR/+*	M	114	68.63 ± 12.39	71	---------	---------	---------
3-1	A	*w/Y; 255B/fruIR; fruIR/+*	M	121	68.02 ± 12.20	69	-0.88	-2.82	0.77
3-1	L	*w/Y; 255B/fruIR; fruIR/+*	M	124	66.23 ± 13.69	67	-3.49	-5.63	0.475
3-1	-	*w; 255B/fruIR; fruIR/+*	F	116	76.37 ± 16.00	79	---------	---------	---------
3-1	A	*w; 255B/fruIR; fruIR/+*	F	120	72.94 ± 11.19	75	-4.49	-5.06	**3.26E-07**
3-1	L	*w; 255B/fruIR; fruIR/+*	F	124	63.72 ± 21.87	65	-16.57	-17.72	**0.002**
6-1	-	*w/Y; 255B/+; +*	M	116	77.52 ± 13.06	78	---------	---------	---------
6-1	A	*w/Y; 255B/+; +*	M	118	77.46 ± 15.82	79	-0.08	1.28	0.125
6-1	L	*w/Y; 255B/+; +*	M	115	76.37 ± 14.50	79	-1.47	1.28	0.361
6-1	-	*w/w1118; 255B/+; +*	F	124	78.97 ± 13.48	79	---------	---------	---------
6-1	A	*w/w1118; 255B/+; +*	F	121	76.71 ± 13.57	81	-2.86	2.53	**0.008**
6-1	L	*w/w1118; 255B/+; +*	F	123	81.28 ± 14.17	83	2.93	5.06	0.118
*Exp 2*									
3-1	-	*w/Y; 255B/fruIR; fruIR/+*	M	88	48.77 ± 26.56	58	---------	---------	---------
3-1	A	*w/Y; 255B/fruIR; fruIR/+*	M	109	61.43 ± 16.27	64	25.95	10.34	**0.024**
3-1	L	*w/Y; 255B/fruIR; fruIR/+*	M	120	53.65 ± 19.56	56	10	-3.45	0.895
3-1	-	*w; 255B/fruIR; fruIR/+*	F	76	68.45 ± 17.05	73	---------	---------	---------
3-1	A	*w; 255B/fruIR; fruIR/+*	F	95	58.04 ± 14.35	62	-15.2	-15.07	**1.55E-15**
3-1	L	*w; 255B/fruIR; fruIR/+*	F	129	53.77 ± 18.60	60	-21.45	-17.81	**1.14E-13**
3-1	-	*w/Y; 255B/CyO; fruIR/+*	M	55	42.18 ± 18.86	46	---------	---------	---------
3-1	A	*w/Y; 255B/CyO; fruIR/+*	M	66	41.52 ± 12.24	44	-1.58	-4.35	0.066
3-1	L	*w/Y; 255B/CyO; fruIR/+*	M	78	42.38 ± 15.69	44	0.48	-4.35	0.536
3-1	-	*w;255B/CyO; fruIR/+*	F	79	73.75 ± 13.13	76	---------	---------	---------
3-1	A	*w; 255B/CyO; fruIR/+*	F	86	57.88 ± 14.28	62	-21.51	-18.42	**0**
3-1	L	*w; 255B/CyO; fruIR/+*	F	101	59.07 ± 13.13	62	-19.9	-18.42	**0**
6-1	-	*w/Y; 255B/+; +/+*	M	121	62.33 ± 18.12	68	---------	---------	---------
6-1	A	*w/Y; 255B/+; +/+*	M	117	57.09 ± 22.64	66	-8.4	-2.94	0.165
6-1	L	*w/Y; 255B/+; +/+*	M	119	62.57 ± 16.22	68	0.39	0	0.478
6-1	-	*w/w1118; 255B/+; +/+*	F	123	75.95 ± 9.37	78	---------	---------	---------
6-1	A	*w/w1118; 255B/+; +/+*	F	122	71.05 ± 13.36	74	-6.45	-5.13	**7.97E-06**
6-1	L	*w/w1118; 255B/+; +/+*	F	124	69.02 ± 12.88	74	-9.13	-5.13	**7.69E-07**
*Exp 3*									
7-1	-	*w/Y; 255B/+; +*	M	124	72.03 ± 18.30	75	---------	---------	---------
7-1	A	*w/Y; 255B/+; +*	M	123	74.18 ± 20.23	80	2.98	6.67	0.151
7-1	L	*w/Y; 255B/+; +*	M	123	71.69 ± 23.51	80	-0.47	6.67	0.171
7-1	-	*w/+; 255B/+; +*	F	124	85.39 ± 25.75	92	---------	---------	---------
7-1	A	*w/+; 255B/+; +*	F	118	83.03 ± 25.60	90	-2.76	-2.17	**0.043**
7-1	L	*w/+; 255B/+; +*	F	119	91.78 ± 19.02	96	7.49	4.35	0.213
1-11 R1	-	*y w sxl/Y; 255B/+; +*	M	25	85.12 ± 14.30	88	---------	---------	---------
1-11 R1	A	*y w sxl/Y; 255B/+; +*	M	45	85.51 ± 19.71	94	0.46	6.82	0.314
1-11 R1	L	*y w sxl/Y; 255B/+; +*	M	76	85.13 ± 13.94	84	0.01	-4.55	0.635
1-11 R1	-	*y w sxl/w; 255B/+; +*	F	49	70.45 ± 31.82	82	---------	---------	---------
1-11 R1	A	*y w sxl/w; 255B/+; +*	F	72	57.39 ± 35.23	76	-18.54	-7.32	**0.011**
1-11 R1	L	*y w sxl/w; 255B/+; +*	F	122	83.31 ± 23.54	86	18.26	4.88	**0.005**
1-11 R2	-	*y w sxl/Y; 255B/+; +*	M	67	91.22 ± 21.02	98	---------	---------	---------
1-11 R2	A	*y w sxl/Y; 255B/+; +*	M	75	75.55 ± 22.66	80	-17.19	-18.37	**2.30E-09**
1-11 R2	L	*y w sxl/Y; 255B/+; +*	M	96	86.31 ± 27.48	98	-5.38	0	0.965
1-11 R2	-	*y w sxl/w; 255B/+; +*	F	69	89.45 ± 25.26	92	---------	---------	---------
1-11 R2	A	*y w sxl/w; 255B/+; +*	F	74	87.32 ± 18.37	88	-2.38	-4.35	0.064
1-11 R2	L	*y w sxl/w; 255B/+; +*	F	121	92.58 ± 17.93	96	3.5	4.35	0.593
11-7	-	*y w sxl/Y; +; +*	M	119	92.76 ± 15.18	98	---------	---------	---------
11-7	-	*y w sxl/+; +; +*	F	120	94.48 ± 18.26	98	---------	---------	---------
10-1	-	*w/Y; 255B/tra; +*	M	124	81.47 ± 14.12	82	---------	---------	---------
10-1	A	*w/Y; 255B/tra; +*	M	122	74.48 ± 13.90	76	-8.58	-7.32	**2.26E-04**
10-1	L	*w/Y; 255B/tra; +*	M	124	79.71 ± 16.97	84	-2.16	2.44	0.293
10-1	-	*w; 255B/tra; +*	F	127	93.40 ± 15.91	96	---------	---------	---------
10-1	A	*w; 255B/tra; +*	F	123	91.38 ± 18.93	98	-2.16	2.08	0.81
10-1	L	*w; 255B/tra; +*	F	126	90.24 ± 22.04	96	-3.39	0	0.732
15-1	-	*w/Y; 255B/tra2; +*	M	125	73.44 ± 16.92	76	---------	---------	---------
15-1	A	*w/Y; 255B/tra2; +*	M	124	75.61 ± 15.06	76	2.96	0	0.469
15-1	L	*w/Y; 255B/tra2; +*	M	125	74.64 ± 21.79	78	1.63	2.63	**0.039**
15-1	-	*w; 255B/tra2; +*	F	124	78.58 ± 25.27	85	---------	---------	---------
15-1	A	*w;255B/tra2; +*	F	125	77.47 ± 27.00	86	-1.41	1.18	0.789
15-1	L	*w;255B/tra2; +*	F	125	79.81 ± 17.45	82	1.56	-3.53	0.144
9-1	-	*w/Y; 255B/+; dsxF/+*	M	74	83.35 ± 22.08	88	---------	---------	---------
9-1	A	*w/Y; 255B/+; dsxF/+*	M	100	34.20 ± 9.96	32	-58.97	-63.64	**0**
9-1	L	*w/Y; 255B/+; dsxF/+*	M	0	---------	---------	---------	---------	---------
9-1	-	*w; 255B/+; dsxF/+*	F	73	87.53 ± 19.27	90	---------	---------	---------
9-1	A	*w; 255B/+; dsxF/+*	F	98	52.00 ± 13.51	52	-40.59	-42.22	**0**
9-1	L	*w; 255B/+; dsxF/+*	F	24	67.33 ± 26.60	78	-23.08	-13.33	**1.87E-04**
14-1	-	*w/Y; 255B/+;dsx/+*	M	124	68.73 ± 21.07	72	---------	---------	---------
14-1	A	*w/Y; 255B/+;dsx/+*	M	124	70.53 ± 17.57	72	2.63	0	0.88
14-1	L	*w/Y; 255B/+;dsx/+*	M	5	46.00 ± 35.30	50	-33.07	-30.56	0.27
14-1	-	*w; 255B/+;dsx/+*	F	123	65.37 ± 28.78	76	---------	---------	---------
14-1	A	*w; 255B/+;dsx/+*	F	123	65.56 ± 30.01	76	0.3	0	0.763
14-1	L	*w; 255B/+;dsx/+*	F	123	73.06 ± 17.51	74	11.77	-2.63	0.735

^*a *^Mean life span, days +/- SD.

**Figure 4:**
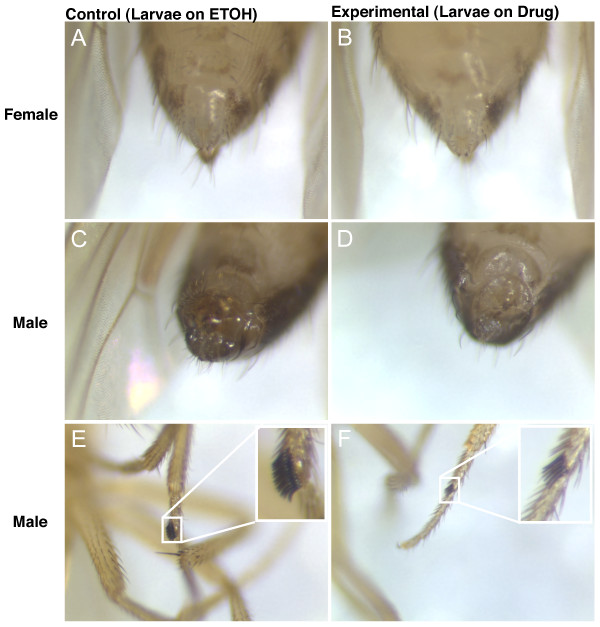
**Effect of *tra *over-expression during development on sexual differentiation of adults**. UAS-*tra *males were crossed to GS255B virgins, cultured on food with drug (+RU486) to drive the over-expression of *tra *during development, or cultured on food with ethanol as the control, as indicated. Pictures were taken at the magnification of 100X. (A, B) Female genitalia. (C, D) Male genitalia. (E, F) Male sex comb.

Conditional over-expression of the pre-mRNA splicing factor gene *tra *during development significantly inhibited sexual differentiation in males, resulting in a lack of much of the external genitalia and a reduction in the size of the sex combs (Figure 4C, D, E, F), however these flies did not exhibit a significant change in life span (Figure 3C; Table 4). In contrast, in females, over-expression of *tra *during development did not detectably affect sexual differentiation (Figure 4A, B), and did not alter life span (Figure 3D; Table 4). Over-expression of *tra *in adults gave no significant change in male and female life span (Figure 3C, D; Table 4). Repeats of the life span assay with *tra *over-expression produced similar results (Table 4, Exp3).

Conditional expression of the *tra*2 RNAi construct in female adults resulted in a small but significant decrease in life span of -12% (Figure 3F). However, expression of the *tra*2 RNAi construct during male and female development or in male adults did not affect life span (Figure 3E, F; Table 4), nor did it have a detectable effect on morphological differentiation.

Over-expression of *dsxM *during development was toxic to males and females and produced only a limited number of adult escapers, while over-expression of *dsxM *in adults caused a small but statistically significant reduction in male and female life span, -5.5% and -7.8%, respectively (Figure 3G, H and Table 4 Exp1); however, this apparent reduction in life span upon over-expression of *dsxM *in adults may not be significant, as it was similar in magnitude to the small variation in life span observed in controls.

Over-expression of *dsxF *during development was lethal to males and produced a limited number of females. This observation is consistent with previous studies where over-expression of DSX during development using a heat shock promoter was found to cause lethality in both males and females [37]. Interestingly, over-expression of *dsxF *specifically in adults dramatically reduced male and female mean life span, by -61.3% and -45.8%, respectively (Figure 3I, J; Table 4, Exp1); and these results were confirmed by repeated experiments (Table 4, Exp3). Similarly, over-expression of the *fru *male isoform A (*fru MA*) during development was lethal, and over-expression of *fru MA *in adults greatly reduced both male and female mean life span, by -61.97% and -64.5%, respectively (Figure 3K, L; Table 4, Exp1).

Developmental or adult-specific expression of one or two inserts of an RNAi construct designed to target the male-specific isoform of FRU [25] did not affect male life span (Figure 3M), but surprisingly, decreased female lifespan significantly (Figure 3N; Table 4). Previous studies where the *Act-GS-255B *driver and various RNAi constructs were used to inactive expression of several genes demonstrates that RNAi *per se *does not affect life span [38,39].

Finally, EP-*sxl*, XP-*dsx*, and XP-*tra2 *gene-over-expression lines were also tested, but did not give a consistent or significant change in life span (< 3% change), when expressed during development or in adults (Table 4, Exp3).

## Discussion

In these experiments, germ line ablation using the *tudor[1]* mutation was examined in a long-lived genetic background, and was found to cause increased mean and median life span in males (+19% and +12%, respectively), whereas female life span was not affected. In a previous report, germ line ablation using the *tudor[1]* mutation increased the median life span of male flies by +8.6% [18]. However, germ line ablation was considered not to have increased life span in that report. Presumably, this was due to a decrease in female life span, and the fact that germ line ablation with a *germ cell-less *mutant failed to extend life span in either males or females. However, *germ cell-less *was reported to have only ~75% penetrance in that study, which conceivably could have masked any life span extension. Here the *tudor *mutation was found to show a greater increase in life span, and overall longer life spans were observed for both control and experimental groups. This is likely due to differences in the genetic background employed: here the relatively long-lived Oregon-R strain was used for crosses to *tudor[1]*, and the control males and control females had median life spans of 82 days and 80 days, respectively (Table 2); whereas in the previous study the relatively shorter-lived Dahomey strain was utilized for crosses, and produced male control median life span of 60 days, and female control median life span of 71 days. In a previous study of *D. subobscura*, "ovariless" females lived significantly longer than virgin female controls or mated female controls [40], and the difference between this and the present results may be due to the difference in genetic background, or the difference in the way or extent of germ line removal caused by specific mutations. In another recent report, elimination of GCs was found to extend median life span by +14% to +78% in males, and by +23% to +100% in females [16]. In those experiments germ line ablation was produced by mis-expression of the *bag of marbles *(*bam*) gene in adult flies, which caused full sterility by day 7 post-eclosion in females, and GC depopulation in the 3^rd ^instar larval (L3) stage or later in males. The presence of a complete germ line in the earlier stages of development followed by GC loss at a later stage could affect life span in a different way, compared with the lack of a germ line from the beginning of embryogenesis, such as is produced by the *tudor *mutation. For example, the *tudor *mutation also partially inhibits formation of the somatic gonad, while ablation of GCs using *bam *mis-expression at later developmental stages allows for complete differentiation of the somatic gonad. Consistent with this idea, in *C. elegans*, extension of life span by ablation of the germ line requires the presence of the somatic gonad [41].

The *Winmodest *program was used to fit the *tudor[1]* mutant data to a Gompertz-Makeham model, which allowed us to separate early mortality from the age-dependent and age-independent mortality. The analysis indicated that germ line ablation in male *Drosophila *extended life span by decreasing the age-independent mortality. That implies that germ line ablation provides a benefit for survival in male flies, and this beneficial effect is constant over the adult life span. This might occur through altered IIS as reported for *C. elegans *hermaphrodites [41].

The other manipulations of the sexual differentiation pathway tested produced either neutral or negative effects on adult fly life span. For example, conditional over-expression of *tra *during development significantly inhibited sexual differentiation in males, yet produced no significant change in adult life span. Expression of *tra *in males has previously been found to feminize locomotor activity patterns [42]. These data demonstrate that it is possible to alter sexual differentiation and perhaps even behavior, at least in males, without necessarily having effects on adult life span.

Over-expression of *dsxF *during development was lethal to males and produced a limited number of females, while over-expression of *dsxF *in adults greatly reduced both male and female life span. Over-expression of *dsxM *during development was toxic to males and females, whereas over-expression of *dsxM *in adults produced only a small, if any, reduction in male and female life span. This indicates that in adults, where sexual differentiation is already complete, changes in expression of specific sex-determination pathway genes are still able to have significant effects on life span.

Interestingly, over-expression of *fru *male isoform A (*fru*MA) during development was lethal, and over-expression of *fruMA *in adults greatly reduced both male and female mean life span. However, expression of an RNAi construct specific for *fruMA *during development or in adults significantly decreased female life span, but did not give a consistent change in male life span. The reason for this effect of *fruMA*-RNAi in females is not yet clear. In females, *fru *P1 transcripts are produced, but not translated [24]. Possibly the *fruMA*-RNAi could still function through *fru *P1 transcripts to affect female life span, or could function through targets other than *fru*.

Taken together with life span results of other assayed genes, our data show little correspondence between the sex-specific mode of action of sexual differentiation genes in development and sex-specific effects of their over-expression or knockdown on adult lifespan. For example, adult-specific over-expression of *fruMA *and *dsxF *shortened life span to a similar extent in males and females. This may be because the sex-specificity of these factors is created during development by means of sex-specific expression, and in our experiments we are forcing expression in each sex, thereby eliminating sex-specificity of action. One possible exception is the developmental lethality caused by over-expression of *dsxF *during larval development, which was complete in males, where it is not normally expressed, and only partial in females, where it is normally present.

Sexual differentiation genes play important roles in germ cell development [20-22]. Therefore, one way that sexual differentiation genes might regulate life span is by modulating germ cell development. Sexual differentiation genes also regulate behavior; for example, *fru *is essential to generate several male-specific behaviors, including male courtship behavior [43]. Although the role of sexual differentiation genes on behavior has not been examined specifically in the adult stage, one way sexual differentiation genes might conceivably affect life span is by altering costly or beneficial behavior patterns in adults. Another promising direction for future experiments will be to look at the interactions between sexual differentiation genes and the pathways and interventions that are known to regulate life span in a sex-specific manner, such as the insulin/IGF1-like signaling (IIS) pathway and dietary restriction (DR) [8,10,11].

## Conclusion

The data demonstrate that manipulation of sexual differentiation gene expression specifically in the adult, after morphological sexual differentiation is complete, is able to affect life span. In addition, by manipulating gene expression during development, it was possible to significantly alter morphological sexual differentiation without a significant effect on adult life span. The data demonstrate that manipulation of sexual differentiation pathway genes either during development or in adults can affect adult life span, sometimes with sex-specific effects, and suggest it should be possible in the future to investigate how the sexual differentiation pathway interacts with specific life span regulatory genes to produce sex-specific differences in longevity.

## Competing interests

The authors declare that they have no competing interests.

## Authors' contributions

DF conducted life span assay of *tudor *mutant offspring. JS and GL conducted experiments with gene over-expression and RNAi. JS performed statistical analyses, with help from DF and GL. JT conceived of and supervised the project, and JT and JS wrote the paper. All authors have read and approved the final manuscript.

## Pre-publication history

The pre-publication history for this paper can be accessed here:

http://www.biomedcentral.com/1471-2318/9/56/prepub
